# Chemoprevention studies within lung cancer screening programmes

**DOI:** 10.3332/ecancer.2015.597

**Published:** 2015-11-24

**Authors:** G Veronesi, A Guerrieri-Gonzaga, M Infante, B Bonanni

**Affiliations:** 1 Division of Thoracic Surgery, European Institute of Oncology, Via Ripamonti 435, Milan 20141, Italy; 2 Division of Cancer Prevention and Genetics, European Institute of Oncology, Via Ripamonti 435, Milan 20141, Italy; 3 Division of Thoracic Surgery, Humanitas Research Hospital, Via Manzoni 56, Rozzano 20100, Italy

**Keywords:** chemoprevention, GGO, lung cancer, screening

## Abstract

While aggressive tobacco control and help to stop smoking are essential weapons in the fight against lung cancer, screening with low-dose computed tomography (LDCT) in high-risk populations and chemoprevention may also contribute to reducing lung cancer deaths. Persons undergoing LDCT screening are an ideal population to be tested for agents potentially able to prevent the development of lung cancer by the regression of precancerous lesions, which are routinely monitored as part of the screening process. Peripheral subsolid nodules appear as particularly suitable targets, since many are adenocarcinoma precursors.

A study on inhaled budesonide (a potential chemopreventive drug) for 1 year found that the mean size of non-solid lung nodules was significantly reduced over 5 years of follow-up, compared to inhaled placebo, in a population of high-risk individuals with indeterminate lung nodules not requiring immediate specific investigation for lung cancer and detected as part of a lung cancer screening program with LDCT.

A new randomised placebo-controlled phase-II trial to test the ability of aspirin to induce the regression of non-solid and partially solid nodules detected by LDCT screening has been started. The effect of aspirin on a miRNA signature able to predict the presence of both cancer and precancerous lesions in high-risk asymptomatic individuals is also being monitored in the trial. This signature was previously shown to predict the presence of both lung cancer and non-solid lung nodules in asymptomatic individuals.

## Introduction

Lung cancer – a disease largely caused by tobacco smoking – is the most common cancer in the world, and the most common cause of cancer death [1]. In most Western countries, tobacco control initiatives have been followed by a smoking reduction and, after a lag, by a reduction in male lung cancer deaths [2]. However, only female lung cancer deaths in some Western countries are declining. In most other countries, lung cancer rates in men and women are increasing in relation to increasing or stabilising smoking prevalence [2].

Efforts to discourage people from smoking and help them to stop once they have started need to be continued. However, early detection and screening programmes for lung cancer using low-dose spiral computed tomography (LDCT) can also help reduce lung cancer mortality [3, 4].

Our research group in Milan started lung cancer screening with LDCT in 2000. A pilot study enrolled 1035 high-risk volunteers. After 10 years of annual scans, most (83%) lung cancers diagnosed were early stage, with 5- and 10-year survival of 64% and 57%, respectively (84% and 65% for stage I); only 12.1% of those operated on had a benign lesion. The Bach model – designed to predict symptomatic cancers – accurately predicted cancer frequency from the third year on, suggesting that overdiagnosis was a minor problem; while our COSMOS risk model – designed to predict screening-detected lung cancers – accurately predicted cancer frequency at baseline and the second screening round [5].

Then, the COSMOS study followed. This study recruited 5203 asymptomatic high-risk individuals in 2004 and 2005 to undergo annual LDCT. After 10 years, a high proportion (77.7%) of the cancers diagnosed were early stage, with a low proportion (14.2%) of false positives, estimated 10% overdiagnosis rate and 82% five-year lung cancer-specific survival [6].

The COSMOS II study started in 2012, recruiting 7500 high-risk individuals from seven centres in Italy. Its principal aims were to validate a serum signature of 13 circulating microRNAs as a predictor of asymptomatic lung cancer [7] and also to validate the COSMOS risk model on an independent cohort. The risk model was designed to select persons for screening and estimate the optimal screening interval for each individual [8].

Another possible approach to reducing lung cancer deaths is chemoprevention. A chemopreventive agent for lung cancer should be able to reverse or suppress the malignant transformation induced in cells of the bronchoalveolar epithelium by carcinogens (known to be present in tobacco smoke). However, the large phase-III trials conducted so far, using putative natural or synthetic chemopreventive agents, have not shown any useful effects, while the administration of beta-carotene and retinoids has proven detrimental [9–12].

Phase-II lung cancer prevention trials have also been disappointing. Most of these focused on the modulation of bronchial dysplasia [13, 14], while no studies were primarily concerned with the peripheral lung where most screening-detected lung cancers occur [3–7]. In fact, peripherally located non-solid lung nodules, as opposed to solid nodules, may often be atypical adenomatous hyperplasia (that may develop into adenocarcinoma) or localised adenocarcinoma [15–19] with the main difference compared to other models that in these cases the difficulty to reach the small lesions for a biopsy and the absence of reliable molecular markers does not allow to have a definitive diagnosis.

## Lung cancer prevention with inhaled corticosteroids

Budesonide is a corticosteroid widely used to treat asthma. It is also a promising chemopreventive compound. In a mouse model, budesonide was found to inhibit all stages of progression of benzo(alpha)pyrene-induced lung tumours [20]. In other mouse models, budesonide delayed the appearance of lung tumours and decreased their growth and progression to carcinoma [21, 22].

The use of budesonide as a chemopreventive agent against lung cancer is also justified by its pharmacodynamic properties. The substance has a short plasma half-life and is selectively retained by airways epithelium. Furthermore, excess budesonide forms esters with long-chain fatty acids, producing a depot of highly lipophilic inactive budesonide within the cell. In a reversible and concentration-dependent manner, intracellular lipases can hydrolyse the esters, releasing the less lipophilic-free budesonide into the cell to increase its retention locally (bronchial epithelium) and prolong its action [23].

Other studies have investigated other steroids. A cohort study on patients with chronic obstructive pulmonary disease (COPD), found that inhaled corticosteroid use (triamcinolone, beclomethasone, flunisolide, or fluticasone) was associated with a dose-dependent reduction in the risk of developing lung cancer [24]. However, a clinical trial of fluticasone versus placebo for 6 months, in persons with bronchial squamous metaplasia or dysplasia found that, while more in the fluticasone arm had a decrease, and fewer had an increase, in number of nodules found on chest CT, in neither case was the difference significant [25].

Lam *et al.* [14] performed a randomised trial to investigate the effect of inhaled budesonide (800 µg twice daily) on 112 heavy smokers (>30 pack-years) with bronchoscopy-identified bronchial dysplasia. Fifty-three were assigned to inhaled budesonide and 53 to inhaled placebo. The main endpoint was a change in the histopathologic grade of dysplasia on bronchial biopsy after 6 months. The secondary endpoint was modification of biomarkers. The study also investigated changes in CT-detected non-calcified lung nodules <8 mm, up to 2 years after treatment.

There were no significant differences in the regression or progression rates of bronchial dysplasia between the groups. Complete response rates were 46% in the budesonide group and 48% in the placebo group (*p* = 0.85), respectively. Bronchial dysplasia progression was also similar: 10% in the budesonide group and 9% in the placebo group (*p* = 0.76). Multiple logistic regression analysis found that smoking was associated with progressive disease, the odds of which increased by 2.5% for each additional pack-year (odds ratio [OR] 1.025, 95% confidence interval [CI] 0.1–5%, *p* = 0.040). Former smokers had a greater probability than current smokers of achieving complete response (OR: 3.03, 95% CI: 1.14–8.09, *p* = 0.027), but this was unrelated to treatment. Sex had a borderline effect on the probability of obtaining complete response, with women 2.3 times more likely than men to achieve complete response (95% CI: 0.9–5.8, *p* = 0.082). Regarding the effect of budesonide on lung nodules, 16/60 nodules (27%) in the budesonide group reduced in size or resolved compared to 14/117 (12%) in the placebo group (*p* = 0.024).

## Randomised trial to assess effect of inhaled budesonide on peripheral lung nodules

Encouraged by findings that inhaled budesonide may have a favourable effect on CT-detected lung nodules, we carried out a double-blind randomised phase-IIb trial (NCT00321893) of inhaled budesonide versus inhaled placebo in persons with indeterminate lung nodules detected by LDCT screening [26].

The trial recruited subjects among the high-risk volunteers already enrolled in COSMOS. LDCT screening provided an opportunity to monitor the effects of budesonide treatment on nodule evolution over the longer term, with the limitation, however, that small nodules that did not qualify for further investigation could not be biopsied and their histological characteristics remained unknown.

To be eligible for the trial, subjects had to have a persistent lung nodule (present on at least two consecutive annual screening scans) of size ≥4 mm, but which did not require further investigation as a suspected cancer according to the COSMOS protocol [26]. Specifically, eligible nodules were one of the following: (a) between 4 and 5 mm maximum diameter, which may or may not have grown; (b) between 5.1 and 8 mm maximum diameter, may or may not have grown, but if grown had a volume doubling time (VDT) of >1 year; or (c) >8 mm, but with negative PET, negative CT with contrast (where feasible), and had VDT >1 year.

The primary endpoint was the effect of budesonide on nodule size in a per person analysis after a year. Secondary endpoints included a lesion-specific analysis (number and size of lesions), changes of tumour markers in sputum and plasma, changes in C-reactive protein, and treatment-related toxicity and safety.

Nodule size changes were assessed according to RECIST criteria [27]. Specifically, for single nodules of longest diameter >5 mm, a reduction in 30% or more in longest diameter was considered a treatment success; for nodules <5 mm, complete disappearance was a treatment success; for multiple nodules, treatment success was complete or partial response according to response evaluation criteria in solid tumours (RECIST) criteria.

Starting April 2006, over 500 COSMOS participants were evaluated, 84% of whom were eligible according to the nodule size/evolution criteria described earlier. All were contacted and about half gave informed consent and received a baseline examination. A total of 202 participants were enrolled over 15 months. Most participants were current smokers, and about 76% were male. Most nodules were between 4 and 5 mm, and about a third were non-solid or partially solid.

The per person analysis (primary endpoint) showed no difference between the budesonide and placebo groups (response rate 2% versus 1% according to RECIST criteria). Analysis according to nodule type and nodule size also showed no differences between the two groups. However a non-significant interaction between treatment effect and nodule type emerged, with a greater effect of treatment in non-solid than partially solid or solid nodules. Furthermore, lesion-specific analysis showed that no target lesions in the budesonide arm underwent size progression, whereas 5% of target lesions in the placebo arm progressed (*p* = 0.02). However, the appearance of new nodules at 12 months did not different between the arms (*p* = 0.41).

Over the year of the study, four lung cancers, and two in each arm were diagnosed. The drug was well tolerated with few side effects, most of which were grade 1 or 2.

We concluded that the non-significant tendency of non-solid and partially solid nodules to regress after a year of budesonide treatment indicated that continued follow-up was warranted since some of these nodules were likely to be adenocarcinoma precursors. Our experience also indicated that only non-solid or partially solid nodules should be followed in future trials, and that RECIST criteria were not adequate for assessing the evolution of small lesions. A study strength was that only stable or slowly progressing nodules were selected (present on two consecutive annual scans), eliminating those likely due to inflammation. In addition to providing an ideal population on which to test new chemopreventive agents for lung cancer, nesting the trial within an LDCT screening study had the advantages of relatively rapid participant accrual and contained costs. The main weakness was that histology was not available for target nodules.

## Continued monitoring of persons recruited to the budesonide trial

We continued monitoring the evolution of lung nodules in the two study arms for 5 years after the end of treatment. We were particularly interested in assessing whether the effect of budesonide on non-solid lesions persisted. Our analysis [28] showed that the mean maximum diameter of non-solid nodules reduced significantly (from 5.03 mm at baseline to 2.61 mm after 5 years) in the budesonide arm, but there was no significant size change in the placebo arm. The mean diameter of partially solid lesions also decreased significantly, but only by 0.69 mm. The size of solid nodules did not change. Neither the number of new lesions nor number of lung cancers differed in the two arms [28]. These long-term findings provide further evidence that budesonide may be useful as a chemopreventive, since some of the nodules that regressed could otherwise have progressed slowly to adenocarcinoma. Despite the absence of definitive diagnosis of target nodules represents a main problem of this model, data from the literature support the notion that many non-solid or subsolid nodules may represent precancerous lesions. In fact, the follow-up of lung nodules among the CT screening in the National Lung Screening Trial indicated that, in contrast to ≥4 mm solid nodules, non-solid nodules were associated with lung cancer risk that progressively increased with increasing time from baseline screening, particularly after 5 years of follow-up [19]. suggesting that at least some non-solid nodules could be lung cancer precursors (rather than invasive carcinoma) destined to develop into invasive carcinomas over an extended period of time. In addition, pathological studies on non-solid nodules resected in association with lung cancer showed that these lesions are frequently atypical adenomatous hyperplasia, adenocarcinoma *in situ*, or adenocarcinoma; [29, 30] however, further studies with larger samples and longer follow-up are required to assess clinical implications of the study results and verify if the shrinkage of nodules size can be correlated with the reduction in cancer risk.

## Rationale for use of aspirin to prevent lung cancer

Long-term use of aspirin has well-documented anticancer properties and appears particularly effective against cancers, including lung cancer, in which chronic inflammation appears to play an etiological role. Recently, published pooled/meta-analyses [31–34] have shown that daily aspirin, taken to prevent cardiovascular events, reduces the incidence of colorectal and other cancers. For example, an analysis of individual patient data from double-blind randomised controlled trials of daily aspirin [34] found that the 20-year risk of cancer death was consistently lower in groups taking aspirin than control groups and that the benefit increased with duration of treatment. The 20-year risk of lung cancer death was reduced by 29% (95% CI: 11–42%) in the aspirin group, a benefit mainly due to an effect against adenocarcinomas and which evident both in smokers and non-smokers.

Several other studies support a protective effect of aspirin against lung cancer. In a prospective cohort study, total NSAIDs use was associated with a small reduction in lung cancer risk, which was strongest for adenocarcinomas in men and long-term former smokers [35]. The Women’s Health Study randomised 39,876 US women aged 45 years or more, with no history of cancer, cardiovascular disease, or other major chronic illness, to either 100 mg of aspirin or placebo and followed them for an average of 10.1 years [36]. While this study found an effect on total cancer or colorectal cancer in women, there was a non-significant reduction in the risk of lung cancer (relative risk 0.78; 95% CI: 0.59–1.03; *p* = 0.08).

A hospital-based case–control study on 868 primary lung cancer cases and 935 hospital controls with non-neoplastic conditions found that lung cancer risk was significantly lower for aspirin users compared to non-users, and prolonged duration of use was also associated with lowered lung cancer risk [37].

The mechanisms by which aspirin reduces cancer risk are unclear [37–43]. Since aspirin and NSAIDs in general protect mainly against cancers in which chronic inflammation seems to play an etiological role, it has been considered that these agents act against cancer by reducing inflammation (by COX inhibition). This is strong evidence that the mechanisms of the chemopreventive effects of NSAIDs are COX-independent [38–40]. The fact that aspirin is effective at low doses also supports this idea, suggesting in fact that the antiplatelet action of NSAIDS is not only responsible for the antithrombotic effect but also antitumorigenic effect [39] although other mechanisms have been proposed [40–43]. Low-dose aspirin may also reduce gastrointestinal bleeding the main side effect of aspirin and most other NSAIDS [42–44].

## Lung cancer chemoprevention trial with aspirin

Based on the above data, and in view of the fact that we are continuing to follow (with annual LDCT) those enrolled in the COSMOS and COSMOS II studies, we have started a trial to investigate the effect of low-dose (100 mg) aspirin on selected lung nodules identified in participants of COSMOS and COSMOS II. Those eligible have non-solid or partially solid lung nodules detected by LDCT, as part of screening, that persist for at least 3 months after initial detection and do not require further diagnostic ascertainment as being suspicious for cancer. Specifically, nodule size is either between 4 and 10 mm with any VDT, or >10 mm with VDT greater than 400 days. Non-target lesions (solid nodules, new lesions, and non-solid or partially solid nodules <4 mm in size) will also be monitored.

Eligible consenting COSMOS participants will be randomised to aspirin (100 mg/day) versus placebo, stratified by trial (COSMOS versus COSMOS II), sex, smoking status (current versus former), and nodule characteristics (non-solid versus partially solid) and treated for one year.

The primary aim is to assess the effect of aspirin in reducing of size and number non-solid and partially solid lung nodules detected by LDCT in person-specific and nodule-specific analyses. Secondary objectives include changes in specific serum microRNAs in relation to aspirin, and correlation of these changes with change in nodule size, change in nodule density, and change in number and size of non-target lesions (including solid nodules). We will also assess response to aspirin according to modified RECIST criteria and monitor of changes in circulating C-reactive protein, urinary cotinine as marker of tobacco exposure, urinary prostaglandin metabolites normalised to urinary creatinine and urinary leukotriene E4, as part of the tolerability assessment.

We need to recruit 128 subjects (64 per arm) to have a 80% power of revealing a hypothesised difference of 0.8 mm in average reduction in nodule size (reduction of 1.0 mm in aspirin group and reduction of 0.2 mm in placebo group) (two-sided two-sample *t*-test, α = 0.05, 1-β = 0.80). The average shrinkage in the placebo arm (−0.2 mm) and standard deviation (1.6 mm) are derived from ad hoc evaluation of our budesonide trial findings.

## MicroRNA markers in aspirin chemoprevention trial

We will assess changes in levels of selected circulating microRNA (miRNA) in relation to aspirin treatment and changes in lung nodule diameter as secondary study endpoints. In 2011, we published a study showing that a test based on the detection of 34 serum miRNAs, identified, with 80% accuracy those with early-stage NSCLCs among a population of asymptomatic high-risk individuals [7]. The same signature also identified (presumably premalignant) non-solid nodules in COSMOS participants (unpublished data). Briefly, we screened 27 individuals with non-solid (ground glass opacity) nodules using our miRNA test, finding that 24/27 (89%) had a ‘positive’ risk score: average 12.5 in this group compared to −2.2 in the group (*N* = 25) with solid nodules, and −4.4 in the group (*N* = 25) without nodules. These differences were significant (<0.001). Thus, the miRNA test is able to predict the presence of non-solid nodules and early-stage lung cancer and has potential for selecting high-risk persons who should undergo screening LDCT; those with lower risk score can avoid LDCT, reducing both screening costs and the harm associated with radiation exposure.

The miRNA test is now available as a fully automatised procedure employing a qRT-PCR array that can screen for the expression of about 800 miRNAs in 300 μl of serum. This will make it possible to assay all miRNAs known to be present in serum. We intend to use this technology to identify serum miRNAs that predict a hypothesised effect of aspirin on non-solid nodules.

## Conclusion

The possibility of using chemopreventive agents to block the progression of precancerous lesions and promote lung tissue repair appears to be a fascinating and promising tool to fight lung cancer. Screening programmes offer ideal cohort populations for testing new chemopreventive agents using innovative intermediate endpoints such as disappearance/reduction of peripheral nodules. A randomised study with Inhalated Budenoside nested in the COSMOS screening study, revealed a new endpoint: peripheral ground glass opacities on LDCT which are likely to be precancerous lesions of adenocarcinoma. These lesions are targeted in an ongoing study using low-dose oral aspirin.

## Conflicts of interest

The authors have no conflicts of interest to declare.
